# Sequence conservation of *Plasmodium vivax* glutamate dehydrogenase among Korean isolates and its application in seroepidemiology

**DOI:** 10.1186/s12936-016-1653-3

**Published:** 2017-01-03

**Authors:** Bomin Seol, Hyun-Il Shin, Jung-Yeon Kim, Bo-Young Jeon, Yoon-Joong Kang, Jhang-Ho Pak, Tong-Soo Kim, Hyeong-Woo Lee

**Affiliations:** 1 Department of Tropical Medicine and Parasitology, Inha University School of Medicine, Incheon, 22212 South Korea; 2 Division of Malaria and Parasitic Diseases, National Institute of Health, Korea Centers for Disease Control and Prevention, Osong, 363-951 South Korea; 3 Department of Biomedical Laboratory Science, School of Public Health, College of Health Sciences, Yonsei University, Wonju, 26493 South Korea; 4 Department of Biomedical Science, Jungwon University, Goesan, Chungbuk 367-805 South Korea; 5 Department of Convergence Medicine, Asan Medical Center, University of Ulsan College of Medicine and Asan Institute for Life Sciences, Seoul, 05505 South Korea

## Abstract

**Background:**

Glutamate dehydrogenase of malaria parasites (pGDH) is widely used in rapid diagnostic tests for malaria. Variation in the *pGDH* gene among Korean isolates of *Plasmodium vivax* was analysed, and a recombinant pGDH protein was evaluated for use as antigens for the serodiagnosis of vivax malaria.

**Methods:**

Genomic DNA was purified from blood samples of 20 patients and the *pGDH* gene of *P. vivax* was sequenced. Recombinant protein was prepared to determine the antigenicity of pGDH by enzyme-linked immunosorbent assay (ELISA).

**Results:**

Partial sequence analysis of the *P. vivax pGDH* gene from the 20 Korean isolates showed that an open reading frame (ORF) of 1410 nucleotides encoded a deduced protein of 470 amino acids. The amino acid and nucleotide sequences were conserved among all the Korean isolates. This ORF showed 100% homology with *P. vivax* strain Sal-I (GenBank accession No. XP_001616617.1). The full ORF (amino acids 39–503), excluding the region before the intron, was cloned from isolate *P. vivax* Bucheon 3 (KJ726751) and subcloned into the expression vector pET28b for transformation into *Escherichia coli* BL21(DE3)pLysS. The expressed recombinant protein had a molecular mass of approximately 55 kDa and showed 84.8% sensitivity (39/46 cases) and 97.2% specificity (35/36 cases) in an ELISA. The efficacy of recombinant pGDH protein in seroepidemiological studies was also evaluated by ELISA using serum samples collected from 876 inhabitants of Gyodong-myeon, Ganghwa County, Incheon Metropolitan City. Of these samples, 91 (10.39%) showed a positive reaction with recombinant pGDH protein. Among the antibody-positive individuals, 13 (14.29%) had experienced malaria infection during the last 10 years.

**Conclusion:**

The *pGDH* genes of *P. vivax* isolates from representative epidemic-prone areas of South Korea are highly conserved. Therefore, pGDH is expected to be a useful antigen in seroepidemiological studies. It was difficult to identify the foci of malaria transmission in Gyodong-myeon based on the patient distribution because of the very low parasitaemia of Korean vivax malaria. However, seroepidemiology with recombinant pGDH protein easily identified regions with the highest incidence of malaria within the study area. Therefore, recombinant pGDH protein may have a useful role in serodiagnosis.

## Background

Microscopic examination is the gold standard method for diagnosis of malaria. Despite the simplicity and low cost, however, it is not always possible to use this method [1]. During the last 20 years, the development of alternative diagnostic methods for malaria, such as rapid diagnostic tests (RDTs), has made it possible to extend biological diagnosis to remote areas that lack advanced medical services. RDTs are lateral-flow immunochromatographic tests that detect specific malaria antigens. They are rapid and simple enough to carry out without electricity, specific equipment, or intensive training of personnel [2–4]. Glutamate dehydrogenase (GDH), an enzyme involved in urea synthesis, catalyzes the reversible oxidative deamination of l-glutamate to form α-ketoglutarate and ammonia, using nicotinamide adenine dinucleotide phosphate (NADP(H)) or nicotinamide adenine dinucleotide (NAD(H)) as cofactor [5]. There are three types of GDH, depending on the cofactor. The enzymes specific for NAD(H) (EC 1.4.1.2) generally catalyze the oxidative deamination of l-glutamate (to generate α-ketoglutarate) and have an alkaline pH optimum, whereas the enzymes specific for NADP(H) (EC 1.4.1.4) usually carry out the reductive amination of α-ketoglutarate (to generate l-glutamate) and have a neutral pH optimum. The third type (EC 1.4.1.3), represented by the vertebrate GDH enzymes, can use both cofactors for the deamination of l-glutamate [6]. *Plasmodium falciparum* contains three genes encoding potential parasite glutamate dehydrogenase (pGDH) proteins; two are found on chromosome 14 (PF14_0164 and PF14_0286, encoding pGDHa and pGDHb, respectively) and one is present on chromosome 8 (PF08_0132, encoding pGDHc) [7, 8]. pGDHa and pGDHb are NADP(H) dependent, and the primary sequence of pGDHb suggests that the protein is located in the apicoplast, whereas the localization and cofactor specificity of pGDHc cannot be predicted. The presence of multiple pGDH proteins is reminiscent of the situation in plants and fungi [9–12]. pGDH is considered integral to the parasite’s antioxidant machinery and is thought to be a potential drug target [8, 13–16]. In recent years, pGDH has been used as an antigen for malaria detection.

In this study, variation of the *pGDH* genes of *P. vivax* isolates from 20 patients living in five malaria epidemic-prone areas of South Korea was investigated, and a recombinant protein was evaluated as a serodiagnostic tool.

## Methods

### Blood sample collection

Patients with clinically suspected malaria who had attended Public Health Centers in Bucheon-si, Gimpo-si, and Paju-si of Gyeonggi Province, Ganghwa County of Incheon Metropolitan City, and Cheorwon County of Gangwon Province, South Korea, were examined for malaria parasites. Blood was collected from symptomatic patients and thin and thick blood smears were prepared for microscopic examination. The usefulness of recombinant pGDH protein in seroepidemiological studies was evaluated using sera collected at Gyondong-myeon, Ganghwa County, Incheon Metropolitan City, in December 2012. The serum samples were stored at −80 °C until use.

### Microscopic examination

Thin blood films were prepared to determine the infectivity of blood samples. The blood films were fixed with methanol and stained with Giemsa stain diluted with buffered water (pH 7.2) to emphasize the parasite inclusions in the red blood cells. The densities of blood-stage parasites were estimated by counting the number of asexual parasites relative to 200 white blood cells (WBCs) and then multiplying the parasite:WBC ratio by 8000 (the assumed number of WBCs per microlitre of blood) [3, 17].

### Amplification of the *pGDH* gene

**Fig. 1:**
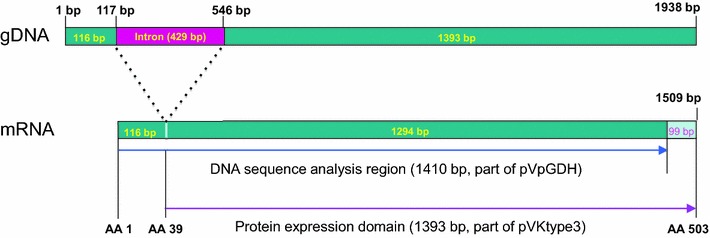
Structure of the plasmid (pVpGDH) used for DNA sequence analysis and expression of the *Plasmodium vivax* parasite glutamate dehydrogenase gene

**Fig. 2:**
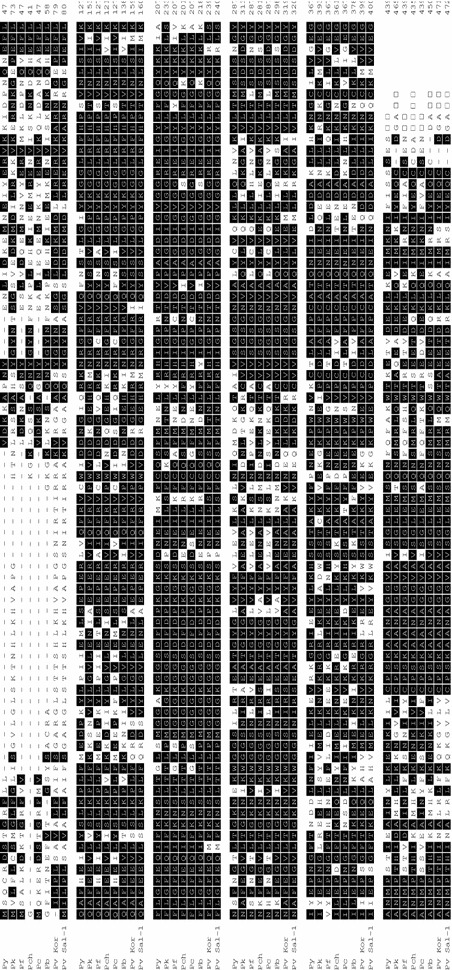
Multiple amino acid sequence alignment of parasite glutamate dehydrogenases among *Plasmodium* species. The deduced amino acid sequence of the type strain of Korean *P. vivax* isolates (Pv Kor, Bucheon strain, GenBank accession No. KJ726751) was aligned with sequences from other *Plasmodium* species. Computer analysis was performed using the multiple sequences alignment program MegAlign. All amino acid sequences were obtained from GenBank using BLAST (http://www.ncbi.nlm.nih.gov). Py, *P. yoelii yoelii* strain 17XNL (XM_719434.1); Pk, *P. knowlesi* strain H (XM_002260680.1); Pf, *P. falciparum* strain FCC1/HN (AY040586.1); Pch, *P. chabaudi chabaudi* (XM_738585.1); Pc, *P. cynomolgi* strain B (XM_004224427.1); Pb, *P. berghei* strain ANKA (XM_673901.1); Pv Sal-1, *P. vivax* strain Sal-1 (XP_001616667)

For amplification of the *pGDH* gene of *P. vivax*, genomic DNA was extracted from each patient’s whole blood using a QIAamp DNA Blood Mini Kit (Qiagen, Hilden, Germany). The polymerase chain reaction (PCR) was performed using TaKaRa Ex Taq DNA polymerase (Takara, Otsu, Japan), 50 ng of purified genomic DNA, and 40 pmol each of forward (PvGDH-F1: 5′-ATT TTA CCC CTC TCG GCC GTG GCC CTT TTC-3′) and reverse primers (PvGDH-R1: 5′-GGC GCC GTC GCA CTG CTC GTA GAT GCT CCT-3′) for DNA sequence analysis (Figs. 1, 2). The PvGDH-Fex1 primer (5′-AGC CAT ATG CGC GCC AAG GTG CGC GGC GC-3′) with an *Nde*I restriction enzyme site (underlined) and the PvGDH-Rex1 primer (5′-GTG CTC GAG CAG GCC GCC CTG CTC CAG GA-3) with an *Xho*I restriction enzyme site (underlined) were used to establish the recombinant protein expression vector. The total PCR volume was adjusted to 50 μL with distilled water. The thermocycling conditions were as follows: denaturation at 94 °C for 5 min; 35 cycles of 30 s at 94 °C, 60 s at 55 °C, and 90 s at 72 °C; and final extension at 72 °C for 5 min. All PCR products were analysed on a 1% agarose gel, confirmed under a UV transilluminator, and purified with a QIAquick Gel Extraction Kit (Qiagen).

### DNA sequencing and analysis

For genotyping of the *pGDH* gene of *P. vivax*, the PCR product of the gene was ligated into a pCR2.1 cloning vector (Invitrogen, Carlsbad, CA, USA) and transformed into *E. coli* TOP10 cells. Cells containing recombinant plasmid were selected on ampicillin-containing medium [18]. The transformants were confirmed by gel electrophoresis of *Eco*RI-digested plasmid DNA prepared with a plasmid isolation kit (Qiagen) according to the manufacturer’s protocol. The *pGDH* gene sequence was determined using the ABI PRISM BigDye Terminator Ready Reaction Cycle Sequencing Kit FS (Perkin Elmer, Cambridge, MA, USA) according to the manual supplied by the manufacturer. The nucleotide and deduced amino acid sequences were analysed using EditSeq and Clustal in the MegAlign program, a multiple sequence alignment program in the DNASTAR package (DNASTAR, Madison, WI, USA). The internet-based BLAST program of the National Center for Biotechnology Information was used to search protein databases.

### Construction of the *pGDH* expression vector

For expression of recombinant pGDH in *E. coli* BL21(DE3)pLysS, the *pGDH* gene was amplified from a blood sample that was confirmed to be infected with the dormant type of *P. vivax*. Amplification was performed as described above with PvGDH-Fex1 and PvGDH-Rex1, which contain *Nde*I and *Xho*I sites at their 5′ ends, respectively. The amplified PCR product was digested with *Nde*I and *Xho*I, purified with a QIAquick Gel Extraction Kit, and integrated into the *Nde*I and *Xho*I sites of the pET28b expression vector (Novagen). The resulting plasmid (named pVKtype3) was subsequently used for the expression of a pGDH-(His)_6_ fusion protein in *E. coli*. The transformants were confirmed both by gel electrophoresis of the plasmid DNA after digestion with *Nde*I and *Xho*I and by DNA sequencing.

### Expression and purification of recombinant pGDH

*Escherichia coli* BL21(DE3)pLysS carrying pVKtype3 was grown to the logarithmic phase in Luria broth containing 50 μg/mL kanamycin, and then expression of the recombinant protein was induced by adding 1 mM isopropyl-1-thio-β-d-galactopyranoside (IPTG) to the culture. The pGDH-(His)_6_ fusion protein was purified by affinity chromatography [19]. The purification was performed under native conditions according to the supplier’s protocol (Novagen). After each purification step, the protein was analysed by sodium dodecyl sulfate polyacrylamide gel electrophoresis (SDS-PAGE) with Coomassie brilliant blue staining.

### Western blot analysis

The recombinant pGDH-(His)_6_ fusion protein was separated on a 12% SDS-PAGE gel and then transferred to a nitrocellulose membrane. After the transfer, the membrane was cut into appropriately sized strips and blocked with 5% skim milk in phosphate-buffered saline (PBS) for 12 h at 4 °C. The membrane was then washed three times for 10 min each with 0.15% Tween 20-PBS. The strips were allowed to react with serum from a patient with malaria or an uninfected patient (diluted 1:100, v/v) for 4 h and then washed as described above. The membrane was subsequently incubated with diluted horseradish peroxidase-conjugated goat anti-human IgG secondary antibody (1:1000, v/v; Sigma) for 3 h at room temperature. For color development, a solution containing 0.2% diaminobenzidine and 0.02% H_2_O_2_-PBS was applied to each well [20, 21].

### Enzyme-linked immunosorbent assay

Enzyme-linked immunosorbent assays (ELISAs) were used to determine whether the blood samples contained antibodies against pGDH antigens of *P. vivax*. Briefly, capture antigen solution (50 μL, 0.5 μg/mL) with recombinant pGDH was placed in a 96-well plate (Corning, Lowell, MA, USA) and incubated for 12 h at room temperature (RT). After aspiration of the antigen solution, the wells were filled with blocking buffer (1% bovine serum albumin, 0.05% Tween 20-PBS) and the plates were incubated for 1 h at RT. The wells were washed three times with 0.05% Tween 20-PBS, and then human serum samples diluted in blocking buffer (1:100, v/v) were added to the wells. Four positive and four negative control serum samples were also added to each plate. After 2 h of incubation at RT, the plates were washed three times with 0.05% Tween 20-PBS, and then horseradish peroxidase-conjugated anti-human IgG (1:2000, v/v; Sigma) diluted in blocking buffer was added. The plates were incubated again for 1 h at RT. The reaction was stopped by washing the plates as described above. The color was developed by adding 100 µL of the peroxidase substrate 2,2′-azino-bis(3-ethylbenzothiazoline-6-sulfonic acid) (Kirkegaard & Perry Laboratories, Gaithersburg, MD, USA) and incubating the plates for 30 min. Optical density was measured at 450 nm, and the cutoff value for positivity was defined as the mean + 2 standard deviations of the negative control samples.

### Calculation of annual parasite incidence

The annual parasite incidence (API) was calculated as the number of malaria-positive patients per 1000 inhabitants for each study area, that is, API = (number of microscopically proven malaria cases/population of administrative area) × 1000.

### Data analysis

Data analyses were performed using GraphPad software (GraphPad Software Inc., La Jolla, CA, USA). A two-tailed *t* test was performed to examine the significance of differences between the malaria patient group and the normal group in the ELISA test. One-way ANOVA followed by a Kruskal–Wallis test was performed to examine the significance of differences between parasitaemia and optical density of ELISA. Pearson’s correlation analysis was performed to examine the relationship between positive rate and API in 2012 and 2013. A *P* value of <0.05 was considered statistically significant.

## Results

### Sequence variation of *Plasmodium vivax pGDH* genes from Korean isolates

The geographical locations of blood sample collection were Ganghwa County (37.31N, 125.33E) of Incheon Metropolitan City; Gimpo-si (37.33N, 126.48E), Bucheon-si (37.29N, 126.46E), and Paju-si (37.88N, 126.76E) of Gyeonggi Province; and Cheorwon County (38.10N, 127.30E) of Gangwon Province. Four blood samples from patients infected with indigenous *P. vivax* were collected from each location during 2010–2011. Amplification of the *pGDH* gene from blood genomic DNA yielded a product of approximately 1400 bp. After purification, the amplified gene fragment was ligated into the pCR2.1 cloning vector (3.9 kb). The plasmid containing the PCR product was named pVpGDH (5.3 kb) and was used for DNA sequence analysis (Fig. 1). Based on the DNA sequencing results, the cloned *pGDH* gene was 1410 bp (excluding the start codon, ATG, and the intron) and encoded 470 amino acids, which were deduced using DNASIS.

**Fig. 3:**
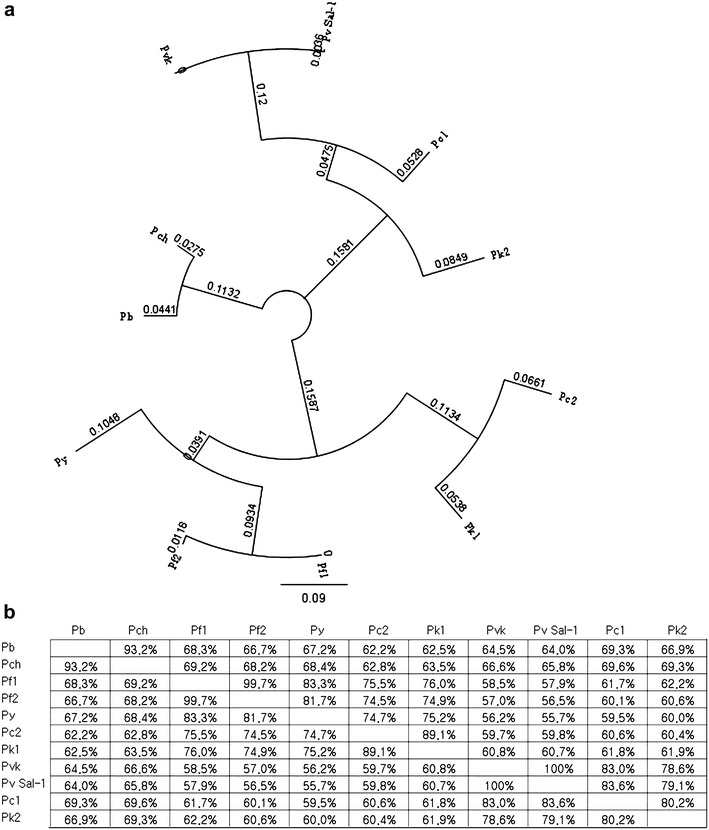
**a** Phylogenetic relationships among parasite glutamate dehydrogenases of several species of *Plasmodium* and **b** percent identity. The deduced amino acid sequence of the type strain of Korean *P. vivax* isolates (Pvk, Bucheon strain, GenBank accession No. KJ726751) was aligned with sequences from other *Plasmodium* species. Computer analysis was performed using the multiple sequences alignment tool of MegAlign. All amino acid sequences were obtained from GenBank using BLAST (http://www.ncbi.nlm.nih.gov). Pb, *P. berghei* strain ANKA (XM_673901.1); Pc1, *P. cynomologi* strain B (PCYB_132800, XM_004224305.1); Pc2, *P. cynomolgi* strain B (PCYB_134020, XM_004224427.1); Pch, *P. chabaudi chabaudi* (XM_738585.1); Pf1, *P. falciparum* (AF269241.1); Pf2, *P. falciparum* strain FCC1/HN (AY040586.1); Pk1, *P. knowlesi* strain H (PKH_131960, XM_002260680.1); Pk2, *P. knowlesi* strain H (PKH_131950, XM_002260679.1); Pv Sal-1, *P. vivax* strain Sal-1 (XM_001616617.1); Py, *P. yoelii yoelii* strain 17XNL (XM_719434.1)

The amino acid and nucleotide sequences were identical to all the Korean isolates (KJ726749–KJ726768). Among the isolates, Pv Kor (Pvk), isolated from Bucheon-si, was designated as the type strain on the basis of *pGDH* gene sequence analysis. The open reading frame showed 100.0% homology with *P. vivax* strain Sal-I (GenBank accession No. XM_001616617.1), 83.0% homology with *Plasmodium cynomolgi* strain B (PCYB_132800, XM_004224305.1), 78.6% homology with *Plasmodium knowlesi* strain H (PKH_131950, XM_002260679.1), 66.6% homology with *Plasmodium chabaudi chabaudi* (XM_738585.1), 64.5% homology with *Plasmodium berghei* strain ANKA (XM_673901.1), 58.5% homology with *Plasmodium falciparum* (AF269241.1), and 56.2% homology with *Plasmodium yoelii yoelii* strain 17XNL (XM_719434.1) (Figs. 2, 3).

### Expression of *pGDH* in *Escherichia coli*

**Fig. 4:**
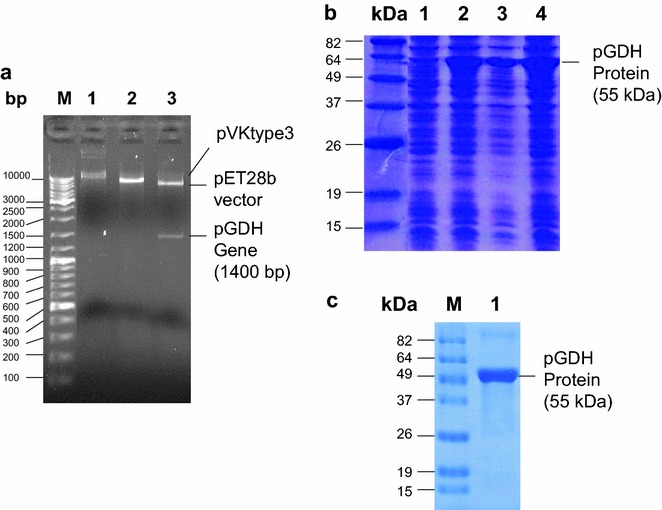
**a** Conformation of the cloned pET28b vector containing the *Plasmodium vivax* parasite glutamate dehydrogenase (*pGDH*) gene (pVKtype3). M, molecular size marker; *lane 1* undigested plasmid; *lane 2 Nde*I-digested plasmid; *lane 3 Nde*I- and *Xho*I-digested plasmid. **b** Expression of the *pGDH* gene in *Escherichia coli* BL21(DE3)pLysS. *Lane M* molecular weight protein marker; *lane 1*, Non-induction clone, *lanes 2–4* induction clones of recombinant pGDH protein. **c** Purification of the pGDH protein from *Escherichia coli* BL21(DE3)pLysS. *Lane M* molecular weight protein marker; *lane 1* purified recombinant pGDH protein

For generation of the expression plasmid, the *P. vivax pGDH* gene sequence from after the intron to before the stop codon was amplified from genomic DNA isolated from blood of an infected patient. The amplified DNA was digested with *Nde*I and *Xho*I and subcloned into the same restriction sites of the pET28b expression vector. The resulting plasmid, pVKtype3, was used to express pGDH fused to a (His)_6_-tag (Fig. 4a). The recombinant plasmid was transformed into *E. coli* BL21(DE3)pLysS and protein expression was induced with IPTG. As evident on the SDS-PAGE gel, the recombinant pGDH protein had a molecular mass of about 55 kDa under native purification conditions (Fig. 4b).

### Antigenicity of the recombinant pGDH protein

**Fig. 5:**
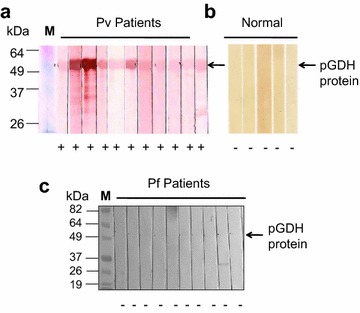
Western blot analysis of recombinant *Plasmodium vivax* parasite glutamate dehydrogenase. **a** Pv patients (*Plasmodium vivax*-infected patients). **b** Normal, healthy volunteers. **c** Pf patients (*Plasmodium falciparum*-infected patients). *M* molecular weight marker

**Fig. 6:**
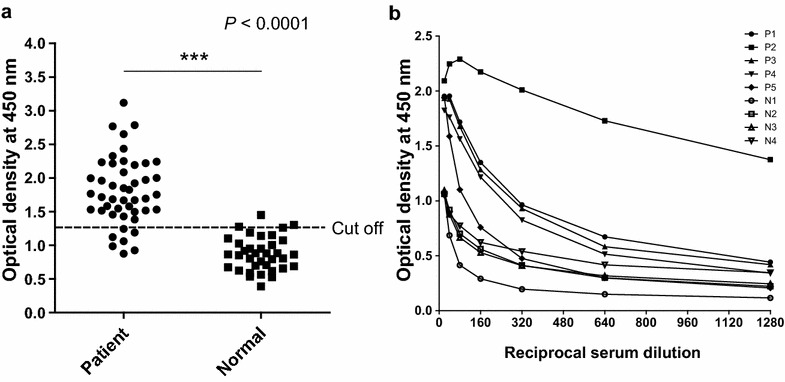
Enzyme-linked immunosorbent assay of immune responses of the vivax malaria patient group and normal individuals to recombinant parasite glutamate dehydrogenase (**a**). Twofold serum dilution of malaria patients (P1–P5) and normal person (N1–N4) (**b**)

**Fig. 7:**
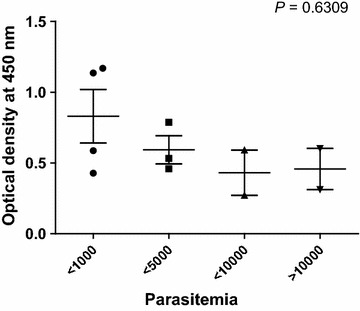
Relationship between parasitaemia and optical density of enzyme-linked immunosorbent assay

The antigenicity of the purified recombinant pGDH protein was assessed by western blot analysis and ELISA. The tested samples contained malaria parasites, as confirmed by microscopic examination, but the parasites had not been counted. Negative sera were collected from volunteer staff from the Korea National Research Institute of Health. All sera of *P. vivax*-infected patients (*n* = X) exhibited a positive reaction with pGDH by western blotting, whereas sera from the normal control group (*n* = 5), who had never been exposed to malaria, tested negative. In addition, pGDH did not react with the sera of *P. falciparum*-infected patients (*n* = 11) (Fig. 5). The antigenicity of the recombinant pGDH protein was then evaluated by ELISA using a larger number of samples. Thirty-nine of the 46 microscopically confirmed malaria-positive serum samples reacted with the recombinant pGDH protein (sensitivity of 84.8%). Only 1 of the 36 samples from the normal control group reacted with the recombinant protein (specificity of 97.2%, Fig. 6a). Positive and negative sera could be differentiated from 1:80 (v/v) serum dilution (Fig. 6b). Parasitaemia decreased the intensity of the reaction, but the difference was not significant (Fig. 7, *P* = 0.6309).

### Usefulness of recombinant pGDH protein in seroepidemiology

**Fig. 8:**
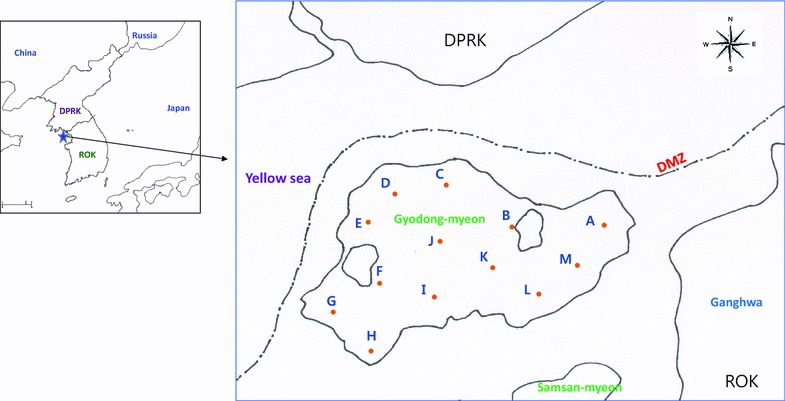
Seroepidemiological study areas of Gyodong-myeon, Ganghwa County, Incheon Metropolitan City. *A* Bongso-ri; *B* Gogu-ri; *C* Insa-ri; *D* Jiseok-ri; *E* Muhak-ri; *F* Nanjeong-ri; *G* Seohan-ri; *H* Dongsan-ri; *I* Yanggap-ri; *J* Samseon-ri; *K* Daeryong-ri; *L* Eumnae-ri; *M* Sangyong-ri. DMZ demilitarized zone; *DPRK* Democratic People’s Republic of Korea; *ROK* Republic of Korea

The usefulness of the purified recombinant pGDH protein was evaluated by ELISA with the serum samples collected from Gyodong-myeon, Ganghwa County, Incheon Metropolitan City, in 2012. A total of 876 serum samples (28.49%) were collected from 3074 inhabitants of 13 villages in Gyodong-myeon (Fig. 8). Ninety-one samples (10.39%) were positive in the ELISA. Nanjeong-ri showed the highest positive rate (16.81%, 19/113), followed by Jiseok-ri (16.39%, 10/61). The lowest positive rate was found in Seohan-ri (2.60%, 2/77) (Fig. 8; Table 1). Among the antibody-positive individuals, 13 (14.29%) had experienced malaria infection during the last 10 years, while 78 (85.71%) had newly acquired antibodies against pGDH. Among the 876 inhabitants, 49 (5.59%) had experienced malaria infection. Nine malaria cases were reported in 2012 and four were reported in 2013; therefore, the API was reduced from 2.93 in 2012 to 1.30 in 2013. Jiseok-ri showed the highest API in 2012 (9.66), in accordance with its high positive rate (16.39%), but the API decreased to 0 in 2013. Nanjeong-ri showed the next highest API in 2012 (7.58), in agreement with its high positive rate (16.81%) in comparison with other villages. Only two villages, Najeong-ri and Samseon-ri, had the API in both years, while the APIs of the other villages were up and down between years. The relationship between the pGDH-positive rate and API was stronger in 2013 than in 2012, although the difference was not significant (Table 1).

**Table 1:** Positive rates of pGLDH-ELISA and annual parasite incidence in study areas

Village	No. of inhabitant	No. of sera tested	No. of positive sera	Positive rate (%)	2012		2013	
					Patient	API	Patient	API
Bongso-ri	167	31	4	12.90	0	0.00	1	5.9
Gogu-ri	308	89	11	12.36	1	3.25	0	0.00
Insa-ri	140	60	3	5.00	1	7.14	0	0.00
Jiseok-ri	207	61	10	16.39	2	9.66	0	0.00
Muhak-ri	138	44	6	13.64	0	0.00	0	0.00
Nanjeong-ri	264	113	19	16.81	2	7.58	1	3.79
Seohan-ri	182	77	2	2.60	0	0.00	0	0.00
Dongsan-ri	140	52	2	3.85	0	0.00	0	0.00
Yanggap-ri	207	82	12	14.63	0	0.00	0	0.00
Samseon-ri	340	105	8	7.62	2	5.88	1	2.94
Daeryong-ri	578	84	8	9.52	0	0.00	1	1.73
Eumnae-ri	214	43	4	9.30	1	4.67	0	0.00
Sangyong-ri	189	35	2	5.71	0	0.00	0	0.00
Total	3074	876	91	10.39	9	2.93	4	1.30

*API* annual parasite incidence

Correlation coefficient between pGLDH-positive rate of 2012 and API of 2012 (r = 0.244, *P* = 0.211)

Correlation coefficient between pGLDH-positive rate of 2012 and API of 2013 (r = 0.318, *P* = 0.145)

## Discussion

pGDH is a target malaria antigen that is widely used to develop monoclonal antibodies for non-microscopic immunochromatographic assays (i.e., RDTs). However, little is known about the ability to detect antibodies to pGDH in serum of patients with malaria. Therefore, the sequence variation of pGDH and its antigenicity was investigated among Korean isolates.

*Plasmodium vivax* has been prevalent in Korea for a long time. However, as the result of a national malaria eradication programme and with help from the World Health Organization, the incidence of vivax malaria rapidly decreased during the 1960s and 1970s [22–25]. Following the report of two cases of malaria in 1985 [25], there were no additional reported cases in Korea until the emergence of one case in 1993 [26] and two cases in 1994 [27]. The incidence of malaria then increased rapidly until approximately 2000 [28]. After that, reported cases of malaria declined again for several years, owing to nationwide efforts to reduce the incidence of this disease. However, malaria has not been eradicated from the Korean peninsula because many travellers and workers come from areas where malaria is prevalent, including North Korea [29]. For these reasons, serological diagnostic tools are needed to support both traditional microscopic diagnosis and antibody testing on a population level to gain an estimate of exposure to malaria in Korea. Currently, the immunofluorescence antibody test (IFAT) is used as the standard serological diagnostic method owing to its high sensitivity. However, the sensitivity of this test can be affected by the training and ability of users [30–32]. Therefore, a new antigen is needed for serodiagnosis. Several recombinant proteins cloned from Korean isolates of *P. vivax* have been tested for use as antigens for serodiagnosis, including Circumsporozoite protein (CSP) subtypes Pv210 [18] and Pv247 [33], merozoite surface protein (MSP) [34], CSP and MSP chimeric proteins [35], aldolase [36], and parasite lactate dehydrogenase [37]. None of these antigens has enabled replacement of the IFAT method because of their comparatively low sensitivity. Therefore, there has been a focus on pGDH. Monoclonal antibodies against pGDH have been used in several RDTs and exhibit a relatively high sensitivity for detection of malaria parasites. However, in the present study the ELISA detected only 84.8% (39/46) of microscopy-positive samples, even though the *pGDH* gene was cloned from a Korean *P. vivax* strain (pVKtype3, Fig. 2). Therefore, antibody detection using the recombinant pGDH protein is not superior to antigen detection using monoclonal antibodies against pGDH. Nevertheless, it was worth investigating whether the recombinant pGDH protein could be used to detect antibodies in asymptomatic patients or symptomatic patients with low parasitaemia (under 50/µL), using antibody detection methods such as ELISA or western blotting. The recombinant pGDH protein was evaluated for its usefulness in seroepidemiology using sera collected from Gyodong-myeon, Ganghwa County, Incheon Metropolitan City, in 2012. This study area is an island consisting of several hills surrounded by rice fields and two large irrigation reservoirs. Serum samples from 28.74% of the inhabitants of this island (876 cases) were tested by ELISA with recombinant pGDH protein as antigen. The villages with a positive rate above 10% are adjacent to the main rice field located in the center of the island and the two irrigation reservoirs, which are potential habitats for anopheline mosquitoes. In contrast, villages with a low positive rate are located in coastal areas and are separated by a hill from the main rice field and the two irrigation reservoirs. In conclusion, the results of this study indicate that antibody detection using recombinant pGDH may provide useful information regarding the prevalence of malaria in certain areas and individuals.

## Conclusion

The findings of the present study suggest that seroepidemiological studies with recombinant pGDH protein, which displayed no amino acid sequence variation among 20 investigated Korean isolates, may be useful for understanding the epidemiology of malaria in Korea.
